# Epidemiological and molecular forensics of cholera recurrence in Haiti

**DOI:** 10.1038/s41598-018-37706-0

**Published:** 2019-02-04

**Authors:** Stanislas Rebaudet, Sandra Moore, Emmanuel Rossignol, Hervé Bogreau, Jean Gaudart, Anne-Cécile Normand, Marie-José Laraque, Paul Adrien, Jacques Boncy, Renaud Piarroux

**Affiliations:** 1 Assistance Publique – Hôpitaux de Marseille, DRCI, Marseille, France; 2 Hôpital Européen Marseille, Marseille, France; 3 Aix Marseille Univ, Marseille, France; 4 Ministry of Public Health and Population, National Public Health Laboratory, Delmas, Haiti; 5 Institut de Recherche Biomédicale des Armées, Département des Maladies Infectieuses, Unité de Parasitologie et d’Entomologie, Marseille, France; 6 Aix Marseille Univ, Institut Hospitalo-Universitaire Méditerranée Infection, VITROME, Marseille, France; 7 Aix Marseille Univ, APHM, IRD, INSERM, SESSTIM, BioSTIC, Marseille, France; 8 Sorbonne Université, INSERM, Institut Pierre-Louis d’Epidémiologie et de Santé Publique, AP-HP, Hôpital Pitié-Salpêtrière, F-, 75013 Paris, France; 9 Ministry of Public Health and Population, Directorate of Epidemiology Laboratory and Research, Delmas, Haiti

## Abstract

Cholera has affected Haiti with damping waves of outbreaks since October 2010. However, mechanisms behind disease persistence during lull periods remain poorly understood. By mid 2014, cholera transmission seemed to only persist in the northern part of Haiti. Meanwhile, cholera appeared nearly extinct in the capital, Port-au-Prince, where it eventually exploded in September 2014. This study aimed to determine whether this outbreak was caused by local undetected cases or by re-importation of the disease from the north. Applying an integrated approach between November 2013 and November 2014, we assessed the temporal and spatial dynamics of cholera using routine surveillance data and performed population genetics analyses of 178 *Vibrio cholerae* O1 clinical isolates. The results suggest that the northern part of the country exhibited a persisting metapopulation pattern with roaming oligoclonal outbreaks that could not be effectively controlled. Conversely, undetected and unaddressed autochthonous low-grade transmission persisted in the Port-au-Prince area, which may have been the source of the acute outbreak in late-2014. Cholera genotyping is a simple but powerful tool to adapt control strategies based on epidemic specificities. In Haiti, these data have already yielded significant progress in cholera surveillance, which is a key component of the strategy to eventually eliminate cholera.

## Introduction

Since October 2010, Haiti continues to experience one of the most aggressive cholera epidemics recorded worldwide since the beginning of the seventh pandemic in 1961^1^. More than 800,000 suspect cases and 9,000 associated deaths have been notified since the epidemic began^2^, although casualties may have been much higher than the numbers reported^3,4^. Following the initial nationwide epidemic explosion^5,6^, cases have progressively receded^2^. However, epidemic rebounds have recurrently affected the Port-au-Prince Metropolitan Area, the northern part of the country and, to a lesser degree, the southwestern tip of the country^7,8^. During epidemic intermissions, Haiti has experienced periods characterized by low levels of cholera incidence periods, as observed in 2014, when an unprecedented lull period in Haiti lasted for over six months^2^. Low-grade cholera transmission appeared limited to the northern part of the country, while the surveillance system recorded zero cases in the Southern Peninsula and only sporadic cases in the Port-au-Prince Metropolitan Area^2^. The lull eventually ended with an acute outbreak that erupted in the Port-au-Prince area in September 2014^2^.

The underlying mechanisms of cholera outbreak recurrence after a cholera lull period have remained poorly understood at a local scale, and several hypotheses may thus be proposed to explain this outbreak in the Port-au-Prince Metropolitan Area. The outbreak may have re-emerged from a local persistence of cholera during the lull period. Conversely, the outbreak may have been caused by the re-importation of cholera from the northern part of the country. As each hypothesis implies a distinct strategy to control cholera during lull periods, understanding this prototypical cholera recurrence event in the Port-au-Prince Metropolitan Area may help to improve cholera elimination strategies worldwide.

Molecular epidemiology of *Vibrio cholerae* strains has contributed to the understanding of cholera dynamics in Asia^9–21^, Africa^22–27^, South America^28,29^ and Haiti. In the latter case, molecular analysis of clinical strains both confirmed the importation of cholera from Nepal in 2010^14,30^ and demonstrated the subsequent clonal differentiation of these *Vibrio cholerae* O1 atypical El Tor strains^31^. On a larger scale, other studies have established a phylogenetic relationship between the epidemics of the current seventh pandemic^32–37^.

We therefore performed an integrated epidemiological and molecular epidemiology study of cholera in Haiti between November 2013 and November 2014. Briefly, routine cholera surveillance data were first analyzed to identify epidemic zones and periods. A subset of *V. cholerae* O1 clinical isolates collected in the country was then randomly selected for genotyping via MLVA (Multiple Loci VNTR [Variable Number of Tandem Repeats] Analysis). Multilocus genotypes (MLGs) were determined and the spatial and temporal distribution of isolate genotypes was analyzed to identify potential strain movement patterns and epidemiological links between cholera foci, with a particular focus on Port-au-Prince. Finally, *V. cholerae* populations corresponding to periods and zones were defined, and population genetics analysis, including fixation indices and assignment tests, was performed to test these links.

## Methods

### Cholera surveillance system in Haiti, and epidemiological data

In 2010, the Haitian Ministry of Public Health and Population (MSPP) established a cholera surveillance system based on the routine daily notification of all suspected cholera cases and associated deaths by up to 416 cholera treatment institutions. According to the WHO standard definition^38^, suspected cases were defined as a patient with acute watery diarrhea, with or without vomiting, and with or without dehydration. Cases and deaths were separately recorded for patients aged ≥5 and <5 years. The Directorate for Epidemiology Laboratory and Research compiled the number of suspected cholera cases and cholera deaths on a national Excel database at a daily and communal scale.

Routine bacteriological confirmation of suspected cholera cases was performed at the National Laboratory of Public Health (LNSP) using standard culture and phenotyping methods^39^. A total of 2,454 stool specimens were cultured between November 2013 and November 2014, out of which 30% were sampled via a sentinel surveillance network of four hospitals in three departments^40^. The remainder was sampled in other cholera treatment centers across the country by the laboratory technicians of each department and medical organizations.

In this study, anonymous MSPP databases of daily reported cases, deaths and stool culture results were used between November 2013 and November 2014. During this period, these data were completed by numerous field investigations performed by epidemiologists from the MSPP, Assistance Publique – Hôpitaux de Marseille (APHM) and UNICEF, in treatment institutions and communities, to confirm or identify cholera outbreaks and determine the underlying risk factors. Their findings were used to help interpret the results of the study.

Country and commune population estimates in 2014 were calculated using 2012 and 2015 population projections of the Haitian Institute of Statistics and Informatics^41^. Satellite estimates of daily-accumulated rainfall (area-averaged TRMM_3B42_daily v7) and mean daily temperature (mean_GLDAS_CLSM025_D_2_0_Tair_f_tavg) were extracted from NASA websites covering the entire surface of Haiti^42^.

### Selection of *V. cholerae* O1 clinical isolates, DNA extraction and MLVA-based genotyping

*V. cholerae* O1 strains isolated at the LNSP were routinely stored at −20 °C in a dedicated biobank. Of the 1,220 isolates collected between November 2013 and November 2014, 275 strains were randomly selected from the laboratory database with commune- and month-specific probabilities calculated beforehand to maximize the spatial and temporal diversity of the sample panel. Among these 275 strains, 191 could be retrieved, successfully subcultured in Haiti and shipped to Marseille, France in Stock Culture Agar vials (Bio-Rad, Hercules, CA, USA) at room temperature. The strains were then recultivated on non-selective Tryptic Soy Agar medium (BD Diagnostic Systems, Heidelberg, Germany) for 24 hours at 37 °C. Suspected *V*. *cholerae* colonies were identified via Gram-staining, oxidase reaction and agglutination assessment with *V*. *cholerae* O1 polyvalent antisera (Bio-Rad).

For DNA isolation, an aliquot of approximately 50 cultured colonies was suspended in 500 μL NucliSENS easyMAG lysis buffer (bioMérieux, Marcy l’Etoile, France). Total nucleic acid was extracted from the *V*. *cholerae* culture samples using a NucliSENS easyMAG platform (bioMérieux) according to the manufacturer’s instructions. The supernatants (100 μL) were then stored at −20 °C for downstream applications^43^.

MLVA-based genotyping of the *V*. *cholerae* isolates was performed using six VNTRs summarized in S1 Appendix. Each VNTR locus was amplified separately. DNA amplification was carried out by preparing a PCR mix containing the following components: 0.375 μL of each primer (20 μM), 1 X LightCycler 480 Probes Master (Roche Diagnostics, Meylan, France) and approximately 100 ng of template DNA. The PCR mix was then brought to a total volume of 30 μL with sterile water. PCRs were performed using a LightCycler 480 System (Roche Diagnostics), with thermal cycling conditions as follows: 95 °C for 5 min; 30 cycles of 95 °C for 30 sec, 58 °C for 30 sec and 72 °C for 45 sec; and 72 °C for 5 min. Aliquots of the PCR products were diluted 1:30 in sterile water. Next, 1 μL of the diluted PCR reaction was aliquoted into a solution containing 25 μL Hi-Di Formamide 3500 Dx Series (Applied Biosystems, Foster City, CA, USA) and 0.5 μL GeneScan 500 LIZ Size Standard (Applied Biosystems). The fluorescent end-labeled PCR amplicons were then separated via capillary electrophoresis using an ABI PRISM 310 Genetic Analyzer (Applied Biosystems) with POP-7 Polymer (Applied Biosystems). Finally, amplicon size was determined using GeneMapper v.3.0 software (Applied Biosystems), and the results were exported to Microsoft Excel for further analysis.

### Temporal and spatial analysis of epidemiological data

Identification of the dry season between November 2013 and November 2014 was computed using temporal scan statistics in SaTScan v9.4.2^44^. A purely temporal retrospective analysis of the daily Haiti-averaged rainfall time series was run with a discrete Poisson model in search for low-rate clusters. Similarly, subdivision of the study window into epidemic periods was computed using SaTScan by searching for low-rate clusters in the time series of daily numbers of suspected cholera cases recorded in Haiti.

As many of the 140 administrative communes lacked cholera treatment institutions and some patients may rather seek treatment outside their living commune, data from several neighboring communes were aggregated after interviewing local health actors and analyzing local reports. The weighted mean coordinates of the 117 merged communes were computed using QGIS v3.0.3^45^, a shapefile of communal administrative boundaries^46^, and a high resolution raster of the population from the WorldPop project^47^. Mapping of data was performed using QGIS v3.0.3 and shapefiles of administrative boundaries and roads provided by CNIGS^46^. Spatial cluster analysis of suspected cholera case distribution for each epidemic period was performed using SaTScan^44^ with a discrete Poisson model. The Port-au-Prince Metropolitan Area hosts over one-third of the Haitian population, and it is the single major communication passage between the northern part of the country and the southern Peninsula. As the main study objective aimed to decipher the origin of the cholera outbreak that hit the capital in September 2014, the 117 merged communes were therefore grouped into three zones before the analyses were carried out: the *PaP* zone (corresponding to the communes of the Port-au-Prince Metropolitan Area); the *North* zone (grouping the northern and eastern communes of Ouest Department as well as the communes of the Artibonite, Centre, Nord-Est, Nord and Nord-Ouest Departments); and the *South* zone (grouping the western communes of Ouest Department as well as the communes of the Sud-Est, Nippes, Sud and Grand’Anse Departments).

### MLVA result analysis

Using VNTR allele sizes, MLGs were identified using excel. A network of MLGs with single-locus variants was assembled using R version 3.2.1 for Mac^48^, and the igraph package. The temporal and spatial distribution of MLGs were illustrated using Excel and QGIS, respectively.

The genetic diversity of the *V. cholerae* O1 populations defined from the identified epidemic periods and the three geographic zones was estimated using the Nei’s index (He)^49^, calculated from allelic frequencies with R and the poppr package. The genotypic diversity (Gd), which corresponds to the probability of drawing two different MLGs in a population, was calculated from genotypic frequencies. Genetic differentiation between populations was assessed using the Wright’s FST fixation index^50^. This was estimated by the Weir & Cockerham θ^51^, which is based on within- and among-population variance components and was computed in FSTAT version 2.9.4^52^. Genetic assignment probability of isolates to previous populations was calculated using the Rannala & Mountain Bayesian algorithm^53^ on GENECLASS2 version 2.0^54^.

The MLG distribution and population genetics analysis findings were confirmed by additional analyses provided as supporting information: comparison between FST and other genetic differentiation indices (S5 Appendix); sensitivity analysis of FST using a different spatial and temporal aggregation of isolates (S6 Appendix); multiple component analysis and hierarchical classification of MLVA results (S7 Appendix); multiple linear regression analysis of genetic, spatial and temporal distances between isolates (S8 Appendix); and Bayesian clustering for spatial population genetics (S9 Appendix).

### Ethical statement

All materials used in the study, including clinical *V. cholerae* strains, were collected by the MSPP during routine surveillance activities to control and prevent cholera. Stool specimens had been obtained from cholera patients with patient and/or legal guardian informed consent. The data were analyzed anonymously with the authorization of the MSPP and in close collaboration with MSPP epidemiologists and biologists. The protocol received authorization from the MSPP National Bioethics Comity (authorization #1415-52). All research was performed in accordance with relevant regulations.

## Results

### Cholera dynamics during the study period

**Figure 1:**
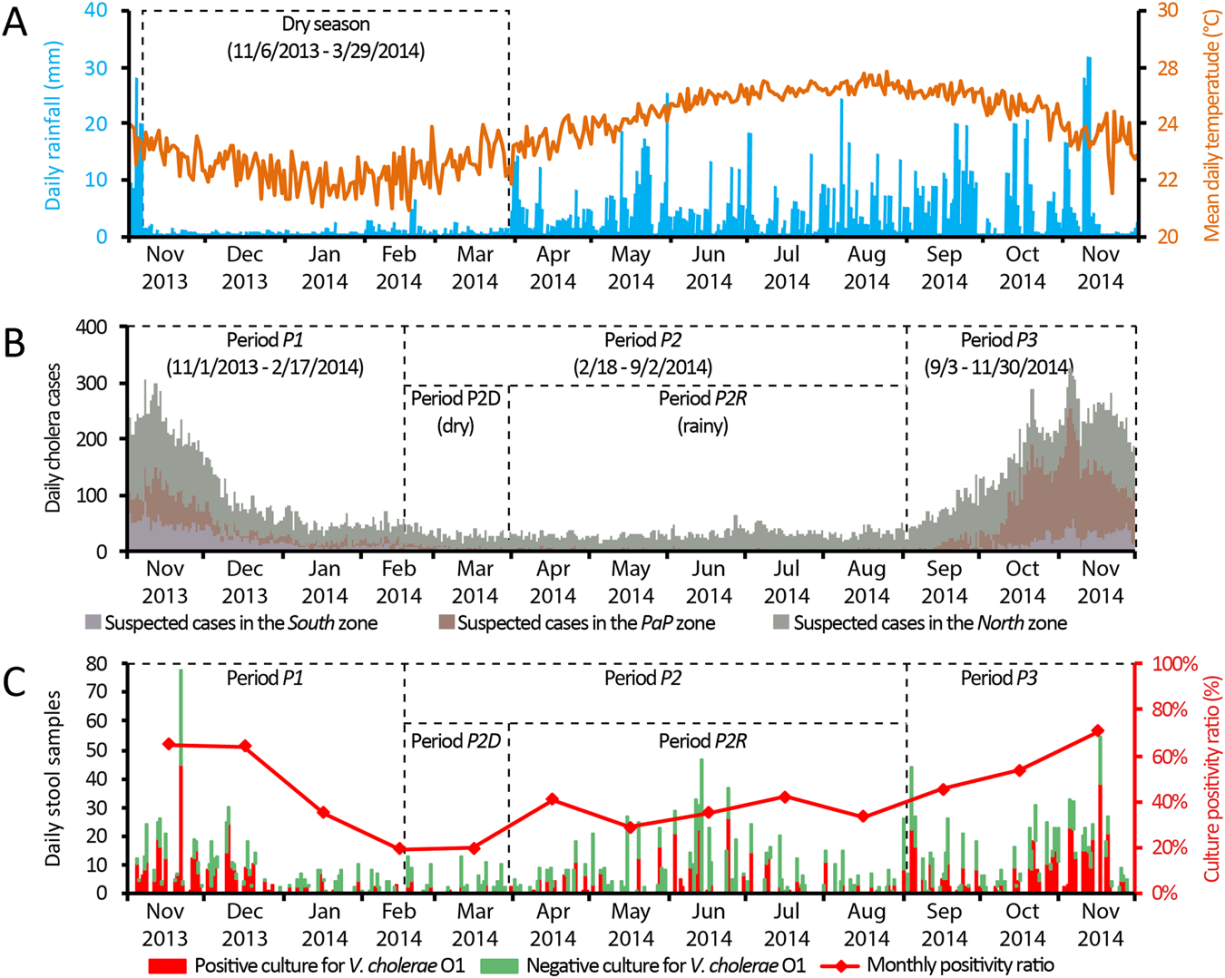
Daily evolution of cholera in Haiti between November 2013 and November 2014: (Panel A) averaged daily accumulated rainfall in Haiti and derived dry season (see S2 Appendix for details); (Panel B) daily number of suspected cholera cases in the *North*, *PaP* (Port-au-Prince) and *South* zones as well as derived epidemic periods identified by the temporal cluster analysis; and (Panel C) daily number of stool samples for culture confirmation of cholera and monthly culture positivity ratio. Period *P1* covers the late 2013 incidence peak and the abrupt decrease in cases during the 2013–2014 dry season; Period *P2* covers a lull phase that extended through the end of the dry season (*P2D*) and the first months of the rainy season (*P2R*); Period *P3* represents the intense epidemic recurrence that started in September 2014.

The study period covered 395 days from November 1, 2013 to November 30, 2014. Based on a temporal cluster analysis of daily accumulated rainfall over Haiti, a significant dry season lasted from November 6, 2013 to March 29, 2014 (Fig. 1), which was the longest dry spell since the beginning of the epidemic (see S2 Appendix for details). Overall, 33,428 suspected cases were recorded by the cholera surveillance system, which corresponded to an average of 2.83 cases/1000 person-years. Forty-nine percent (95%-confidence interval, 47–51) of the 2,415 collected stool samples yielded *V. cholerae* O1 growth upon culture. Specific characteristics of the three spatial zones of the country, *North*, *PaP* and *South*, are summarized in S3 Appendix.

**Figure 2:**
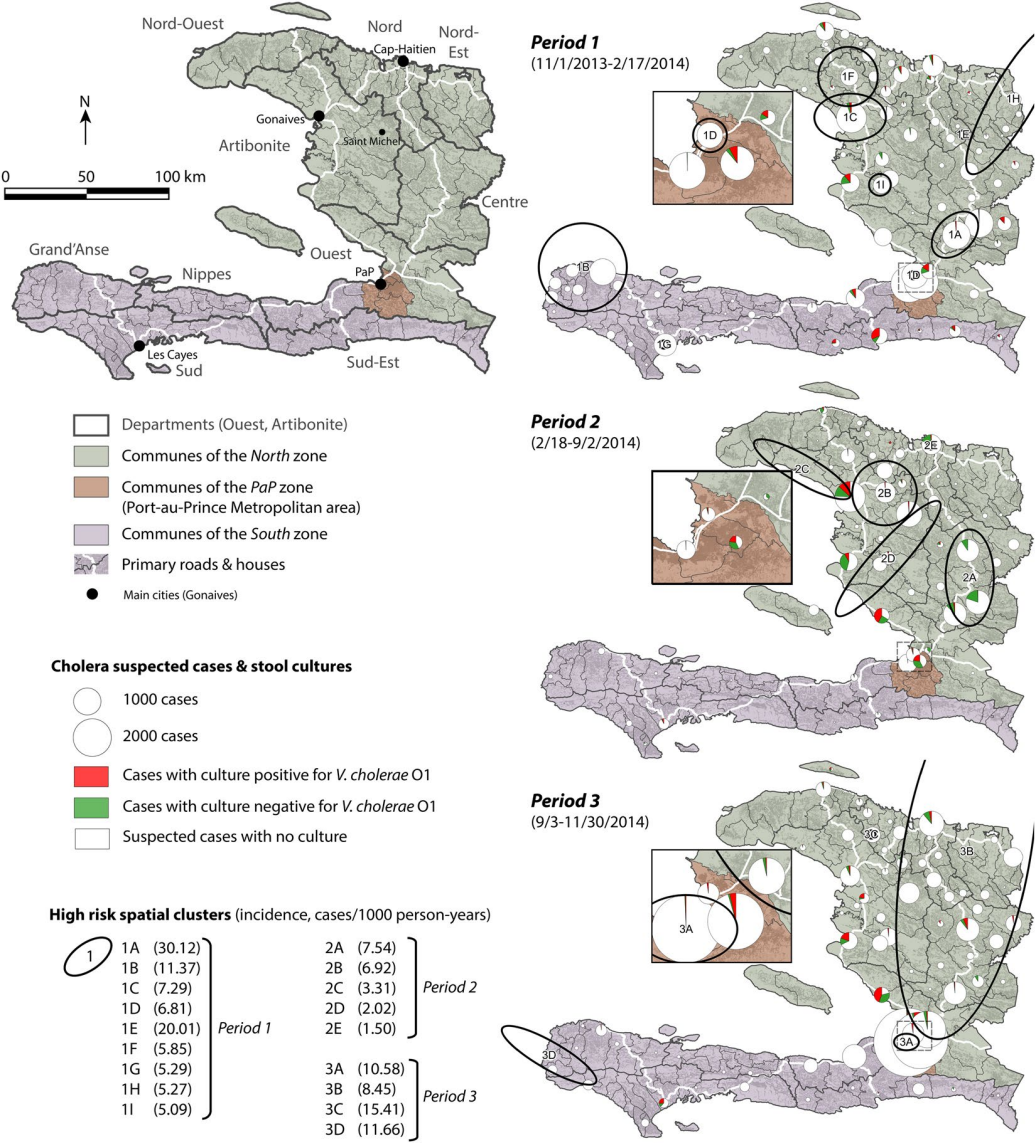
Situation map and communal distribution of cholera in Haiti between November 2013 and November 2014: number of suspected cholera cases; proportion of stool sampling and culture results; and high-risk spatial clusters. The situation map shows the three zones of the study in green, brown and purple, the 10 departments, primary roads and main cities and towns cited in the text. For each of the three periods identified by the temporal cluster analysis (Fig. 1), pie charts represent the number of suspected cholera cases notified by each commune grouped into culture-positive cases, culture-negative cases and non-sampled cases. Black ellipses exhibit significant high-risk spatial clusters for each period.

Temporal cluster analysis of daily suspected cases revealed three distinct epidemic periods (Fig. 1, S4 Appendix). Period *P1* (11/1/2013-2/17/2014) included the end of the late-2013 incidence peak and the abrupt decrease in cases during the 2013–2014 dry season. An average of 3.81 cases/1000 person-years was recorded, and the culture positivity ratio was 58% (54–62). Cases were clustered in the *North* zone (in the Artibonite, Centre and Nord-Est Departments), the *PaP* zone (Port-au-Prince Metropolitan Area) and the western tip of the *South* zone (Grand’Anse Department) (Fig. 2), although cases rapidly receded from most foci.

Period *P2* (2/18-9/2/2014) was a lull phase, which extended over the last 40 days of the dry season (*P2D*) and the first five months of the 2014 rainy season (*P2R*). Only 1.08 cases/1000 person-years were recorded, with no significant difference between the dry and rainy sub-periods (Fig. 1 and S4 Table). The culture positivity ratio during period *P2* was only 34% (31–37), although it represented a significant increase from a markedly low 19% (11–27) during the dry sub-period *P2D* to 35% (32–39) after the return of the rainy season. Cases primarily retracted in residual foci in Artibonite Department (notably Gonaives, the most affected commune, and Saint Michel) and Centre Department (e.g., Hinche, Mirebalais and Lascahobas) (Fig. 1 and Fig. 2). Cholera was apparently extinct in the *South* zone. The *PaP* zone recorded only 572 suspected cases (0.39 cases/1000 person-years) during period *P2*, and 41% of the 87 cultures tested positive for *V. cholerae* O1 during period *P2* (Figs 1, 2 and S3 Table).

Period *P3* (9/3-11/30/2014) corresponded to an intense epidemic recurrence with 5.70 cases/1000 person-years, which started in early September during the rainy season and peaked at over 300 cases per day on November 5, 2014. The positive culture ratio increased to 59% (55–63) during this period. Many cases clustered in a vast area of the *North* zone and the western end of the *South* zone (Fig. 2). Most importantly, nearly half of the 14,923 suspected cases recorded significantly clustered in the *PaP* zone (Fig. 1), with 10.11 cases/1000 person-years. In the *PaP* zone, the epidemic exploded in the Martissant region after case numbers had increased in Corail and Cité Soleil for nearly two months with no epidemiological surveillance. However, the origin of this epidemic recurrence remained unknown.

### Stool sampling and MLVA results

During the 13-month study period, stool cultures for cholera confirmation were performed on 7% of suspected cases throughout the country (S4 Appendix). Significantly more effort was focused on culture confirmation during the lull period *P2* than during periods *P1* and *P3*. However, this disproportion was corrected in our genotype analysis, as the number of complete MLGs was not significantly different between the three periods (S4 Appendix). Of the 275 randomly selected *V. cholerae* clinical isolates, 177 isolates were successfully genotyped via MLVA, including three isolates with mixed infections by more than one *V. cholerae* O1 clone. Overall, 178 complete MLGs were reconstructed.

**Figure 3:**
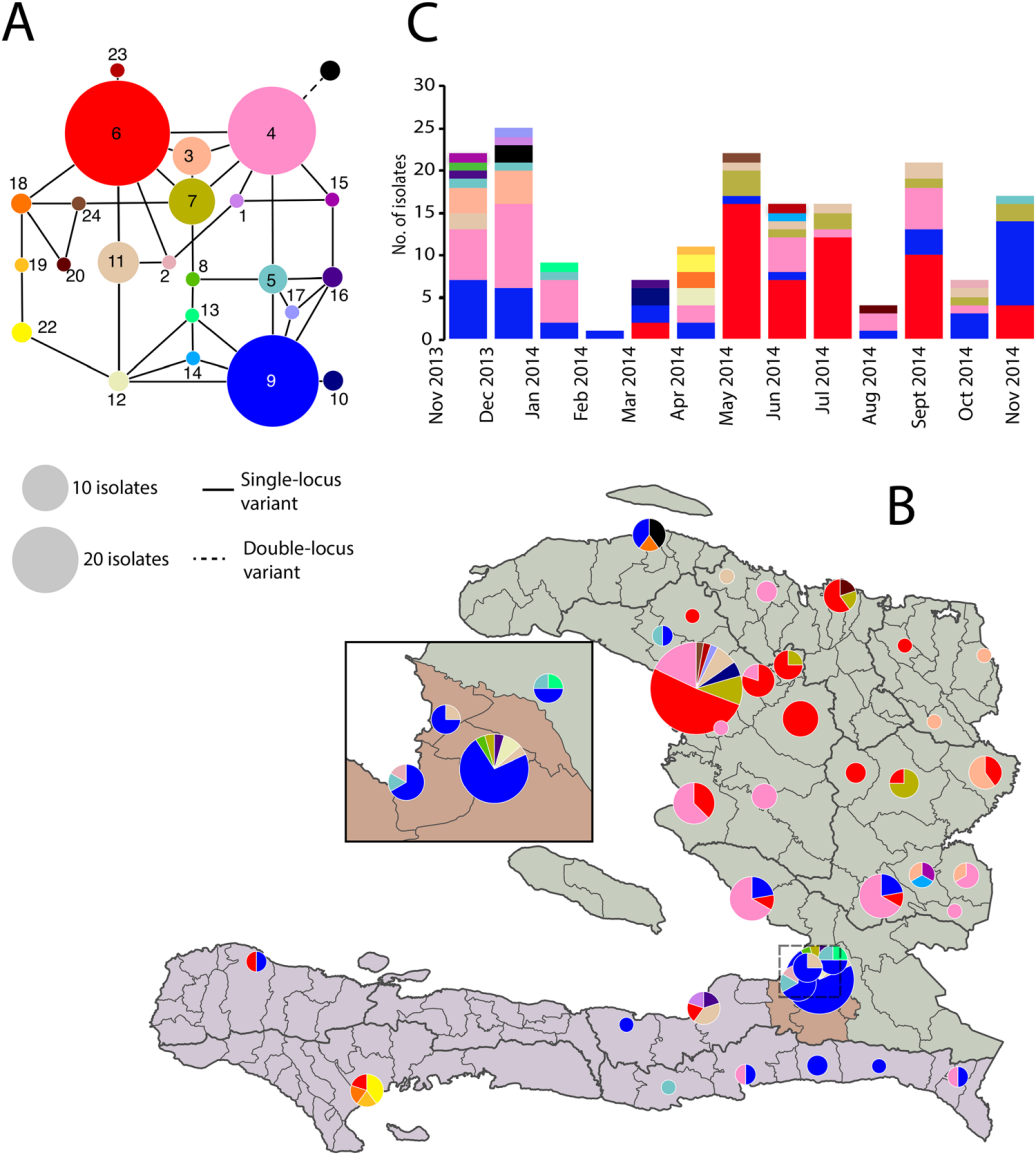
MLVA-based multilocus genotypes (MLGs) of *V. cholerae* O1 clinical isolates in Haiti between November 2013 and November 2014: (Panel A) clonal complex, (Panel B) spatial distribution and (Panel C) temporal distribution. Each color represents a unique MLG. The size of the nodes in the network (Panel A), the size of pie-charts on the map (Panel B) and the height of stacked histograms on the graph (Panel C) are proportional to the number of isolates. Solid edges in the network (Panel A) represent single-locus variants between two MLGs (i.e., allele difference at one of the six loci), and dashed edges double-locus variants (i.e., allele difference at two of the six loci).

According to the results, the VC9 microsatellite locus was monoallelic, while the VC1, VC4, VC5, LAV6 and VCMS12 assays revealed two, five, two, six and three allelic variants, respectively. The observed genetic diversity (Nei’s index) of the entire population was 0.273. MLVA identified 24 closely related MLGs: all but one formed a network of single-locus variants (Fig. 3 Panel A). Three frequent MLGs – #4 (pink), #6 (red) and #9 (blue) – were identified in 126 (71%) of 178 isolates.

### Spatial and temporal distribution of multilocus genotypes

MLGs #9 and #4 were frequently isolated throughout the study period, while other major MLGs (such as MLG #3) were only detected during the initial months. MLG #6 and MLG #7, two single-locus variants of MLG #4, appeared later during the study period (Fig. 3, Panel C). MLG #5 seemed to temporally disappear during period *P2*. Several other MLGs (e.g., #1, #2, #8, #13, #14 and #15) that were closely related to the main MLGs were only isolated once during the study (Fig. 3, Panels B and C). MLG distribution also exhibited a strong spatial pattern: 34 of the 36 MLG #4 isolates, 41/51 of MLG #6, 7/7 of MLG #3 and 9/10 of MLG #7 were located in the *North* zone, while 23 of the 39 MLG #9 were isolated in the *PaP* zone (Fig. 3, Panel B). MLGs #19 and #22 were only isolated in the *South* zone.

Consequently, the most frequently detected genotypes in the *North* zone were MLGs #*4*, #*6*, #3 and #7. MLG *#6* likely emerged from MLG #4 in Gonaives during the dry season. It successfully multiplied locally during the second part of period *P2*, then expanded in Artibonite Department (notably Saint Michel) to the rest of the *North* zone and the *South* zone (Table 1 and Fig. 3). Similarly, MLG #7 likely emerged in Gonaives from MLG *#6* or MLG #4 during period *P2* before spreading to the nearby regions.

**Table 1:** Genetic diversity (Nei’s He index) and differentiation (fixation index FST) of *V. cholerae* O1 populations in Haiti between November 2013 and November 2014.

Period-based populations	No. isolates	Diversity		FST							
		Gd	He	*P2*	*P3*						
*P1*	57	0.763	0.242	0.213*	0.061*						
*P2*	79	0.731	0.270		0.084*						
*P3*	42	0.735	0.238								
**Zone-based populations**	**No. isolates**	**Diversity**		**FST**							
		**Gd**	**He**	***PaP***	***South***						
*North*	125	0.763	0.208	0.499*	0.220*						
*PaP*	32	0.471	0.148		0.150*						
*South*	21	0.830	0.326								
**Period and zone-based populations**	**No. isolates**	**Diversity**		**FST**							
		**Gd**	**He**	***North2***	***North3***	***PaP1***	***PaP2***	***PaP3***	***South1***	***South2***	***South3***
*North1*	38	0.691	0.208	0.304*	0.149*	0.421*	0.489*	0.336	0.202*	0.522*	0.095
*North2*	64	0.623	0.152		0.028	0.671*	0.689*	0.577*	0.505*	0.505*	0.002
*North3*	23	0.691	0.212			0.507*	0.552*	0.394	0.296	0.399	−0.169
*PaP1*	6	0.278	0.111				−0.042	−0.027	0.049	0.604	0.431
*PaP2*	10	0.460	0.093					0.009	0.110	0.649*	0.516
*PaP3*	16	0.500	0.194						−0.028	0.510	0.236
*South1*	13	0.722	0.233							0.464*	0.108
*South2*	5	0.720	0.267								0.174
*South3*	3	0.444	0.333								

*P1*, *P2* and *P3* correspond to the *V. cholerae* O1 populations of the three periods. *North1* represents the population from the *North* zone during *P1, etc*.

Gd, genotypic diversity; He, Nei’s diversity index; FST, Weir & Cockerham fixation index.

* significant FST.

By contrast, the most frequent genotype in the *PaP* zone remained MLG #9: 5 of 18 isolates during period *P1*, 7 of 18 isolates during *P2*, and 11 of 18 isolates sampled during the epidemic of period *P3* (Table 1 and Fig. 3). While MLG #9 was present in the three zones during period *P1*, it appeared to retract in the *PaP* zone during the lull period *P2*, before expanding again to the *North* and *South* zones during *P3* (Table 1 and Fig. 3).

### Genetic diversity and structure of *V. cholerae* populations

Population genetics statistical analyses based on allele frequencies showed that the diversity of *V. cholerae* O1 isolates remained stable between November 2013 and November 2014 (Table 1), even during the lull period *P2*, which interestingly did not exhibit any decrease in genetic diversity (Table 1). However, the differentiation index FST showed a marked temporal structure between *P1* and *P2* (Table 1). Spatially, the genetic diversity of *V. cholerae* clinical isolates appeared greater in the *North* and *South* zones than in the *PaP* zone (Table 1). Additionally, *PaP* isolate populations exhibited a strong genetic differentiation with the *North* population (FST index = 0.499, Table 1).

Combining the three periods and the three zones, high genetic differentiation was observed between the *North1* (isolates from period *P1* in the *North* zone) and *North2* populations, while FST was low between *North2* and *North3*. FST indexes remained very high between the *North* and *PaP* zones throughout the study period. Conversely, no significant genetic differentiation was observed between *PaP1*, *PaP2* and *PaP3*. Although too few isolates were collected in the *South* zone to perform significant population genetics comparisons, *South2* appeared differentiated from all other populations.

FST appeared highly correlated with three other genetic differentiation indices: D (Host), GST (Nei) and GST (Hedrick) (S5 Appendix).

### Assignment of *V. cholerae* isolates during epidemic recurrence in Port-au-Prince

**Figure 4:**
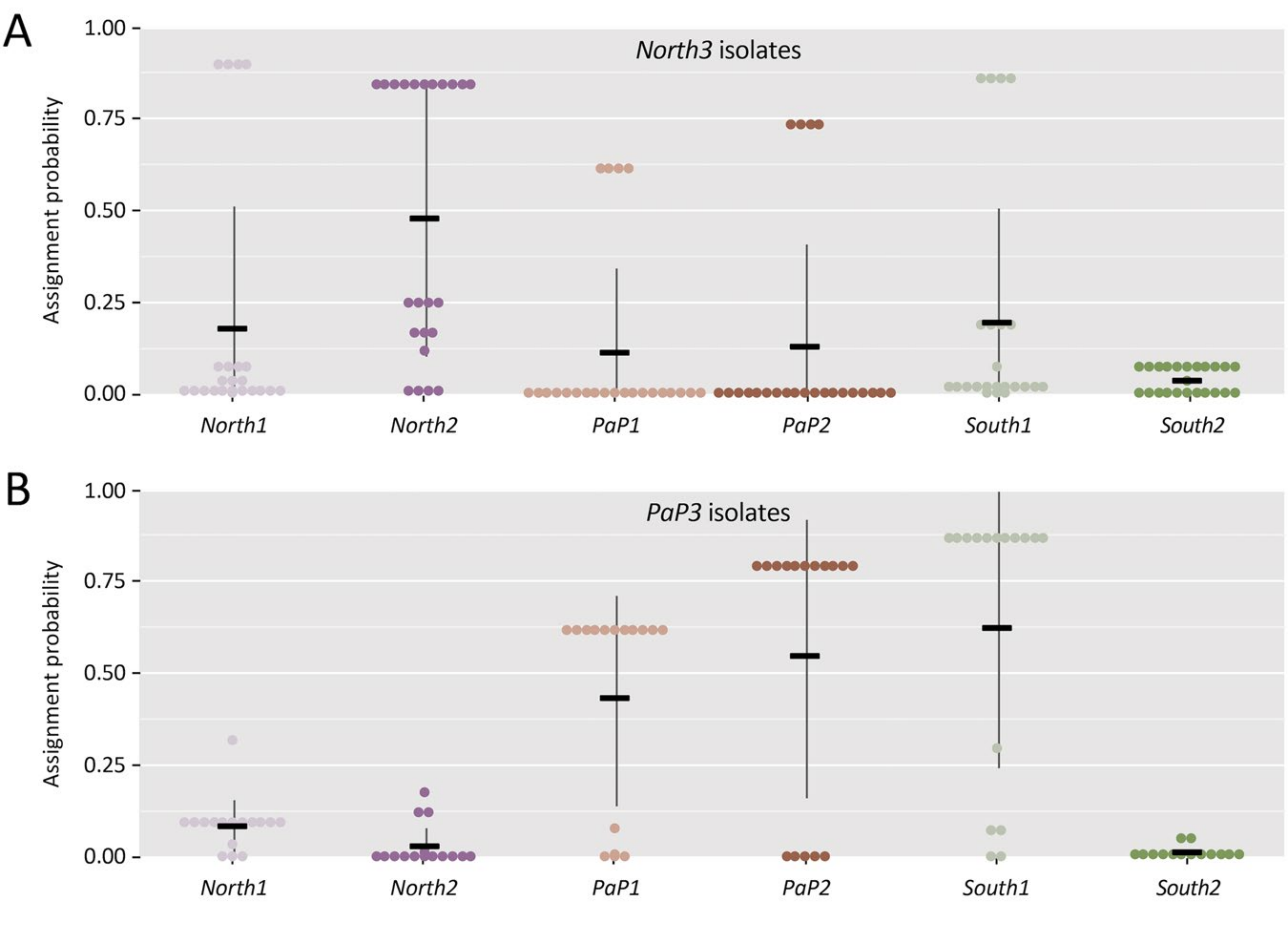
Assignment probabilities of North3 (Panel A) and *PaP3* (Panel B) isolates to previous populations. Dots represent the assignment probability of each isolate of the analyzed population to each of the other populations. Black bars represent the average assignment probability of the analyzed population to the other populations, with standard errors.

The origin of the outbreak that started in September 2014 was assessed using assignment analysis of isolates collected during period *P3*. Most of the 16 isolates sampled in the *PaP* zone during the period *P3* (*PaP3* isolates) were preferentially assigned to the previous local populations *PaP1* and *PaP2* (Fig. 4, Panel B). Conversely, probability of assignment to the previous populations *North1* and *North2* was very low (Fig. 4, Panel B). Additionally, no major difference was observed in the assignment probabilities of *PaP3* isolates between the populations *PaP1* and *PaP2*. Assignment of *PaP3* isolates to the *South1* population was strong, while it was negligible to the *South2* population.

Assignment of *North3* isolates was higher with the *North2* population, although some isolates probably originated from the *PaP2* population. The *South3* population comprised only three isolates that were likely imported from the *North2* or *PaP2* populations.

## Discussion

Cholera incidence in Haiti exhibited a dramatic reduction during the very dry period of late 2013. During several months of lull period, the disease appeared nearly extinct, especially in the *South* zone and in the Port-au-Prince area where only a few sporadic confirmed cases occurred. Following the return of rainfall in April 2014, the national positivity ratio of stool culture for *V. cholerae* O1 gradually increased, before the epidemic resurged in September mainly in the Port-au-Prince Metropolitan Area.

Our genotyping results confirm the intense diversification of the *V. cholerae* O1 clone that was introduced in Haiti in 2010^31,55^. The temporospatial distribution of genotypes (MLG) suggests two different mechanisms of cholera persistence in the northern part of the country and the Port-au-Prince Metropolitan Area. The *North* zone appeared affected by oligoclonal outbreaks of various genotypes, which either rapidly vanished or successfully persisted over time, exhibiting a metapopulation pattern (*i.e*., a group of connected local populations). Despite the low incidence in this area, genotypic diversity was significant and represented a source of cholera spread across the country, as previously described^56^. Conversely, cholera in the *PaP* zone showed little genetic diversification. A few autochthonous genotypes persisted during the marked lull period, before causing the subsequent epidemic peak in September 2014. This acute outbreak in *PaP* was thus likely not re-imported from persisting foci in the *North* zone. Instead, it rather likely re-emerged locally, by a mechanism that our study was not designed to determine more precisely. Some authors have stated the existence of established environmental reservoirs of cholera in Haiti^55,57–59^. However, isolation of toxigenic *V. cholerae* O1 in Haitian surface waters has remained sporadic^55,57–62^, and usually concomitant to local cholera cases, persisting rainfall and widespread open defecation and laundry in the rivers^63^. Such conditions render it difficult to determine whether *V. cholerae* O1–positive surface waters constitute perennial reservoirs at the source of cholera outbreaks or only transient vectors of cholera following contamination by nearby infected patients^57^. The outbreak in *PaP* could also have come out from a low-grade persistent interhuman transmission of the disease. Indeed, considering the number of suspected cholera cases recorded by the MSPP during the lull period *P2* in the *PaP* zone, the likelihood of additional suspected cases undetected by the surveillance system, and the concomitant positivity ratio of stool cultures for *V. cholera* O1, the number of “true” symptomatic cholera patients may theoretically have been sufficient to sustain continuous chains of interhuman and peridomestic transmission (S3 Appendix).

As suggested by a previous study, MLVA results may have been marginally influenced by the sub-culturing process^64^. In addition, our population genetics analysis was limited by the partial sampling of cholera isolates. Indeed, many communes never sent stool specimens for cholera culture confirmation, mainly for logistic reasons, as was the case in Grand’Anse Department. Furthermore, strain availability in the LNSP biobank also reflected the cholera dynamics: samples were very scarce in the *South* zone during Period *P2* because suspected cases were sporadic. This may explain why our results did not exhibit any significant bottleneck during the lull period^55^. We tried to optimize the representativeness of our strain panel by randomly selecting isolates in a way that maximized the spatial and temporal range, so that only 9% of our isolates originated from the four sentinel sites. Additionally, FST and assignment probabilities were also partly biased by the fact that the large outbreak in Port-au-Prince was oligoclonal. However, we did not expect that the *PaP3* population would be almost exclusively composed of genotypes already present in *PaP2* but not in the *North2* population. Although we cannot be sure that these genotypes did not circulate outside *PaP* during *Period P2* before returning to cause the outbreak during *Period P3*, our analyses found this to be a less probable scenario than local persistence of low-grade transmission cases. Finally, our results may have been influenced by the partitioning of our isolate population. The spatial division in *PaP*, *North* and *South* was determined by the theoretical objective of the study (which was to assess the origin of the late 2014 outbreak in the Port-au-Prince Metropolitan Area) and by the fact that the capital is a required crossroad between the northern part of the country and the Southern Peninsula. The temporal division was obtained by cluster analysis. We thus conducted a sensitivity FST analysis using an alternative time and space aggregation of isolates according to their sampling trimester and department, which exhibited the same structure pattern (S6 Appendix). We also performed a multiple component analysis and hierarchical classification of MLVA results; this grouped the isolates into three genetic clusters, for which distribution confirmed the marked genetic differentiation between the *North* and *PaP* zones (S7 Appendix). A similar pattern was obtained when analyzing the relationship between genetic, spatial and temporal distances between isolates using multiple linear regression (S8 Appendix). Finally, a Bayesian clustering algorithm for spatial population genetics without assuming predefined populations exhibited a remarkable match with our chosen populations (S9 Appendix).

Although such biases have been a concern in most of the retrospective molecular epidemiology studies dealing with cholera in resource-constrained countries, such challenges did not prevent researchers to draw definite evidence concerning the origin of other cholera epidemics as well as the overall dynamics of the seventh cholera pandemic^33,36,37^. Whole genome sequencing (WGS)-based phylogenic analysis is a powerful tool that could help minimize such sampling biases. However, MLVA has been shown to reflect the same genetic relatedness of isolates as WGS in several cholera studies^65,66^ and at a much lower cost. Additionally, due to the slow mutation rate of the core genome of *V. cholerae*^33^, WGS may not have provided a sufficient resolution over the short period and narrow space of our study.

Despite these limitations, we believe that the persistence of cholera in the northern part of Haiti and the Port-au-Prince Metropolitan Area during the 2014 lull period may be attributed to two distinct weaknesses in the cholera elimination strategy^67^ and the nationwide cholera rapid-response strategy that was implemented in mid-2013^68^. In the *North* zone, outbreaks were well detected but not effectively controlled. Response teams in charge of targeting cholera-affected communities with awareness campaigns, soap distribution and water chlorination had only been implemented from July 2013 onward. Response interventions remained slow and few in number during this period^68^. By contrast, epidemiological surveillance in the *PaP* zone appeared deficient, as gradually increasing local transmission remained undetected and unaddressed, especially in unsafe neighborhoods, such as Cité-Soleil, or in rapidly expanding and disorganized slums in the north of the Port-au-Prince area, such as Corail (authors personal data from field investigations). Following the epidemic recurrence of September 2014, cholera spread towards the Southern Peninsula and the northern departments.

Remarkably, cholera incidence remained low during five months after the onset of the 2014 rainy season. Although our study was not designed to analyze mechanisms underlying this extended lull, we believe this may be due to the combination of several factors: (1) a low cholera incidence at the end of the 2013–2014 dry season, (2) a relatively dryer rainy season in 2014 compared to previous years (S2 Appendix); and (3) the implementation of the nationwide cholera rapid-response strategy^68^. Although this response strategy was not able to eliminate cholera during the 2014 lull period, we believe it may have mitigated the effect of rainfall on transmission during a few months, until the outbreak finally exploded in the Port-au-Prince.

Our results remain relevant today and highlight the necessity to improve both the quality of epidemiological cholera surveillance^69^ and the effectiveness of the struggle to limit cholera transmission in Haiti. Four years later, suspected cholera cases records have become more exhaustive in Haitian treatment institutions, even though cholera surveillance has still not been extended to the community level, as planned by the 2016–2018 mid-term development of the national plan for cholera elimination by the Haitian government^70^. Thanks to important logistical efforts, stool sampling between January and August 2018 has been performed for 81% of the 2,961 reported suspected cholera cases, and culture results are becoming more often considered to better target active transmission foci and prevent cholera dissemination. In the current context of a new remarkable lull in cholera transmission in Haiti^2^, this progress in surveillance is becoming critical in order to promptly identify and target every single and last case, to eventually reach the goal of cholera elimination in Haiti by the planned deadline of 2022^67^.

## Supplementary information

Supporting information

Supplementary Dataset

## Data Availability

All data generated or analyzed during this study are included in this published article (and in Supplementary Dataset file).
