# Supplementary material for: Epidemiological and molecular forensics of cholera recurrence in Haiti
Source: Sci Rep. 2019 Feb 4;9:1164. doi: 10.1038/s41598-018-37706-0 (PMC6361935; doi:10.1038/s41598-018-37706-0)
Supplement: Supplementary file 1 — Supporting information [file 41598_2018_37706_MOESM1_ESM.pdf]

Epidemiological and molecular forensics of cholera recurrence in Haiti

Supporting information

Author list:

Stanislas Rebaudet, Sandra Moore, Emmanuel Rossignol, Hervé Bogreau, Jean Gaudart, Anne-Cécile Normand, Marie-José Laraque, Paul Adrien, Jacques Boncy, Renaud Piarroux

S1 Appendix: List of analyzed VNTRs

S1 Table – Characteristics, primer sequences and PCR conditions of the six VNTRs

Locus name	Repeated pattern	Chr.*	Position †	Primer sequence (5'→3')
VC1	AACAGA	1	137106	fw: CGGATACTCAAACGCAGGAT<br>rv: 6FAM-CTTTCGGTCGGTTTCTCTTG
VC4	TGCTGT	2	187759	fw: TGTTTGAGAGCTCGCCTCTT<br>rv: PET-TCATCAAGATGCACGACACA
VC5	GATAATCCA	1	1915539	fw: AGTGGGCACAGAGTGTCAAA<br>rv: VIC-AATTGGCCGCTAACTGAGTG
VC9	GACCCTA	1	467111	fw: CGTTAGCATCGAAACTGCTG<br>rv: NED-AGAAAACAATCGCCTGCTTG
LAV6	ACCAGA	2	303939	fw: NED-GCCTCCTCAGAAGTTGAGAATC<br>rv: CCGATGAACTCTCTGAACTGG
VCMS12	TTTTGAT	1	1568189	fw: VIC-GAGGTCTAGAATCTGCCCGA<br>rv: AAGCGCTGTGGGTAGAAGTG

* Chr.: chromosome.

† Based on the reference strain El Tor N16961 (GenBank accession numbers: AE003852.1 and AE003853.1).

Composition of PCR mix: 0.375 µL of each primer (20 µM), 1 X LightCycler 480 Probes Master (Roche Diagnostics) and approximately 100 ng of template DNA. PCR mix brought to a total volume of 30 µL with H_2O.

Thermal cycling conditions: 95°C for 5 min; followed by 30 cycles of 95°C for 30 sec, 58°C for 30 sec and 72°C for 45 sec; 72°C for 5 min.

S2 Appendix: Identification of dry seasons between 2010 and 2014

Dry seasons between the beginning of the epidemic in October 2010 and the end of the study period in November 2014 were identified using temporal scan statistics on SaTScan v9.4.2 [1]. A temporal analysis of the daily Haiti-averaged rainfall time series was performed with a discrete Poisson model in search of low-rate clusters. A separate analysis was run for each period between early October and late September of the following year. Characteristics of the identified dry seasons are summarized in S2 Table. The 2013-2014 dry season was the longest of the four dry seasons since October 2010.

We also identified and characterized rainy seasons (S2 Table). The 2014 rainy season was among the shortest, and was the less wet of the four rainy seasons since 2011. It also was slightly warmer than previous ones.

S2 Table – Characteristics of dry and rainy seasons between October 2010 and November 2014

Dry season	Start date	End date	Duration (days)	Cumulated rainfall in Haiti (mm)	Mean daily rainfall in Haiti (mm)	Mean daily temperature in Haiti (mm)
2010-2011	11/7/2010	4/1/2011	146	257	1.8	21.8
2011-2012	10/16/2011	3/14/2012	151	158	1.0	22.4
2012-2013	10/27/2012	4/17/2013	173	202	1.2	22.8
2013-2014	11/6/2013	4/29/2014	175	234	1.3	22.6
**Rainy season**	**Start date**	**End date**	**Duration (days)**	**Cumulated rainfall in Haiti (mm)**	**Mean daily rainfall in Haiti (mm)**	**Mean daily temperature in Haiti (mm)**
2011	4/2/2011	10/15/2011	197	1428	7.2	24.3
2012	3/15/2012	10/26/2012	226	1448	6,4	24.6
2013	4/18/2013	11/5/2013	202	1357	6,7	24.7
2014	4/30/2014	11/13/2014	198	1117	5,6	24.8

S3 Appendix: Comparison of the three spatial zones characteristics

As the main study objective aimed to decipher the origin of the cholera outbreak that hit the capital in September 2014, the 117 merged communes were grouped into three zones: the *PaP* zone (corresponding to the commune of the Port-au-Prince Metropolitan Area); the *North* zone (grouping the northern and eastern communes of Ouest Department as well as the communes of the Artibonite, Centre, Nord-Est, Nord and Nord-Ouest Departments); and the *South* zone (grouping the western communes of Ouest Department as well as the communes of the Sud-Est, Nippes, Sud and Grand'Anse Departments). The overall characteristics of the three zones were summarized in S3 Table.

S3 Table – Comparison between study zones characteristics

	Entire country	All zones			All periods in zone *PaP*		
		North	*PaP*	*South*	*PaP1*	*PaP2*	*PaP3*
No. Communes	140	78	7	55	7	7	7
No. merged communes	117	68	3	46	3	3	3
Population, x1,000 inhab.	10,911819912	5,424145	2,723538724	2,764136	2,7242723538	2,7242723538	2,7242723538
Mean daily accumulated rainfall (mm)	3.7	4.3	3.7	2.7	1.2	3.7	4.9
Cumulated no. suspected cholera cases	33428	19210	9696	4522	2514	572	6610
Cumulated incidence (cases/1000 person-years)	2.83	3.27	3.29	1.51	3.09	0.39	9.95
No. stool samples for cholera culture	2237	1579	348	228	110	87	151
Proportion of cases with result of confirmation culture	7%	8%	4%	5%	4%	15%	2%
Positivity ratio of stool cultures for V. cholerae O1, % (CI95%)	49% (47-51)	42% (39-44)	64% (59-69)	69% (63-75)	66% (58-75)	41% (31-52)	75% (69-82)
No. multilocus genotypes (MLGs)	178	125	32	21	6	10	16

During the lull period *P2*, the MSPP recorded 572 suspected cholera cases in the *PaP* zone, a stool culture was performed for 15% of them, and 41% (CI95%, 31-52) of cultures grew positive for *V. cholerae* O1 (S3 Table). Considering the likelihood of additional suspected cases undetected by the surveillance system, the number of "true" symptomatic cholera patients may thus have exceeded 235 (177-297) over 197 days. Considering a median incubation period of 1.4 days (95%CI, 1.3-1.6) [2], these patients may theoretically have been sufficient to sustain continuous chains of interhuman and peridomestic cholera transmission.

S4 Appendix: Comparison of epidemic period characteristics

The characteristics of the identified epidemic periods were summarized and compared using Kruskal-Wallis rank sum test for count data indicators (rainfall, cases, stool samples cultures and genotypes) and using Fisher's exact test for proportions (suspected cases with confirmation culture, and culture positivity ratio). Indicators and *p*-values are presented in S4 Table.

S4 Table – Comparison between study period characteristics

		All periods				Period *P2*		
	Entire period	*P1*	*P2*	*P3*	*p*-value	*P2D* (Dry)	*P2R* (Rainy)	*p*-value
Start date	1/11/13	1/11/13	2/18/14	3/9/14		2/18/14	3/30/14	
End date	11/30/14	2/17/14	2/9/14	11/30/14		3/29/14	2/9/14	
No. days	395	109	197	89		40	157	
Mean daily accumulated rainfall (mm)	3,7	1,4	4,0	5,7	<0.0001	0,9	4,8	<0.0001
Cumulated no. suspected cholera cases	33428	12225	6280	14923	<0.0001	1247	5033	0.54
Cumulated incidence (cases/1000 person-years)	2,88	3,81	1,08	5,70	<0.0001	1,06	1,09	0.54
No. stool samples for cholera culture	2237	592	886	759	<0.0001	100	786	0.54
Proportion of cases with result of confirmation culture	7%	5%	14%	5%	<0.05	8%	16%	0.25
Positivity ratio of stool cultures for V. cholerae O1, % (CI95%)	49% (47-51)	58% (54-62)	34% (30-37)	59% (55-62)	<0.0001	19% (11-27)	35% (32-39)	<0.0001
No. multilocus genotypes (MLGs)	178	57	79	42	0.3	6	73	0.1

Comparison between rainfall, cases, samples and MLGs was performed using Kruskal-Wallis rank sum test.
Comparison between proportions of culture and positivity ratios was performed using Fisher's exact test for count data.

S5 Appendix: Comparison between FST and other genetic differentiation indices

To ensure reliability of our population genetics results, we compared FST estimated by the Weir and Cockerham θ [3], with three other common differentiation indices: the indexes D (Host) [4], GST (Nei) [5], GST (Hedrick) [6].

The pairwise comparisons between FST and other indices point out strong linear relationships (S5 Figure). This confirms our results regardless of the index selected.

We initially chose FST because this index is widely used in the literature and facilitates informative comparisons, and because debates on possible biases in certain situations neither take away the interest of this marker in assessing population structure, nor seem to us to be conclusive [4,7–14].

S5 Figure – Correlation between FST and other differentiation indices

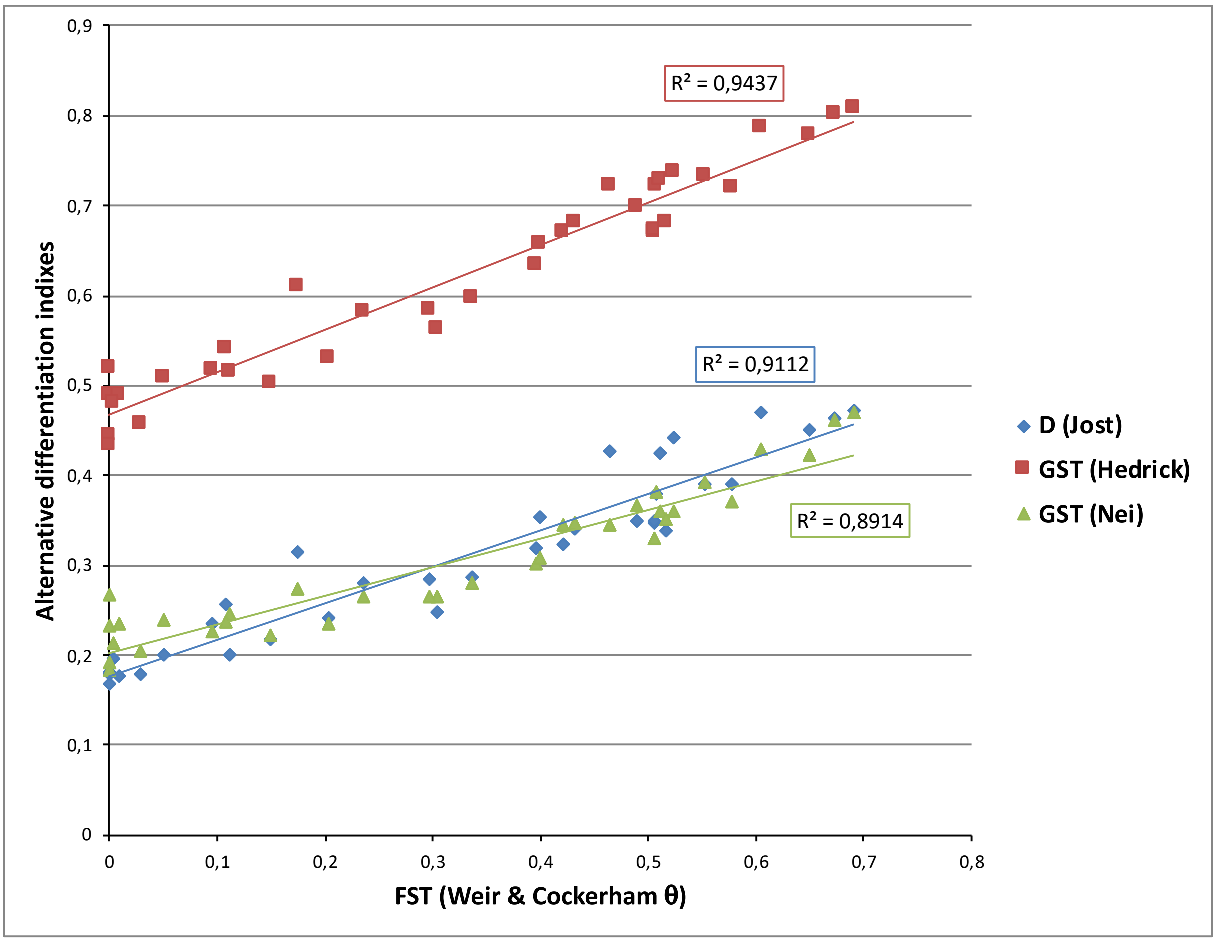

S6 Appendix: Sensitivity analysis of fixation index FST with a different spatial and temporal aggregation of isolates

To ensure that the identified genetic structure of the 178 cholera isolates was not artificially generated by the analyzed populations, we conducted a sensitivity FST analysis using an alternative time and space aggregation, and FSTAT version 2.9.4 [15]. Instead of periods *P1 P2* and *P3*, we temporally aggregated isolates according to their sampling trimester (*T1*, Oct 2013 – Dec 2013; *T2*, Jan 2014 – Mar 2014; *T3*, Apr 2014 – Jun 2014; *T4*, Jul 2014 – Sept 2014; *T5*, Oct 2014 – Dec 2014) (S6 Figure Panel C). Instead of zones *North PaP* and *South*, we spatially aggregated isolates according to their sampling department (*DSO*, Ouest; *DSA*, Artibonite; *DSC*, Centre; *DSN*, Nord; *DSNE*, Nord-Est; *DSNO*, Nord-Ouest; *DSSE*, Sud-Est; *DSNI*, Nippes; *DSS*, South; *DSGA*, Grand'Anse) (S6 Figure Panel B).

S6 Figure – Alternative aggregations of *V. cholerae* O1 clinical isolates in Haiti between November 2013 and November 2014: (Panel A) clonal complex of MLVA-based multilocus genotypes (MLGs), (Panel B) distribution by department and (Panel C) distribution by trimester.

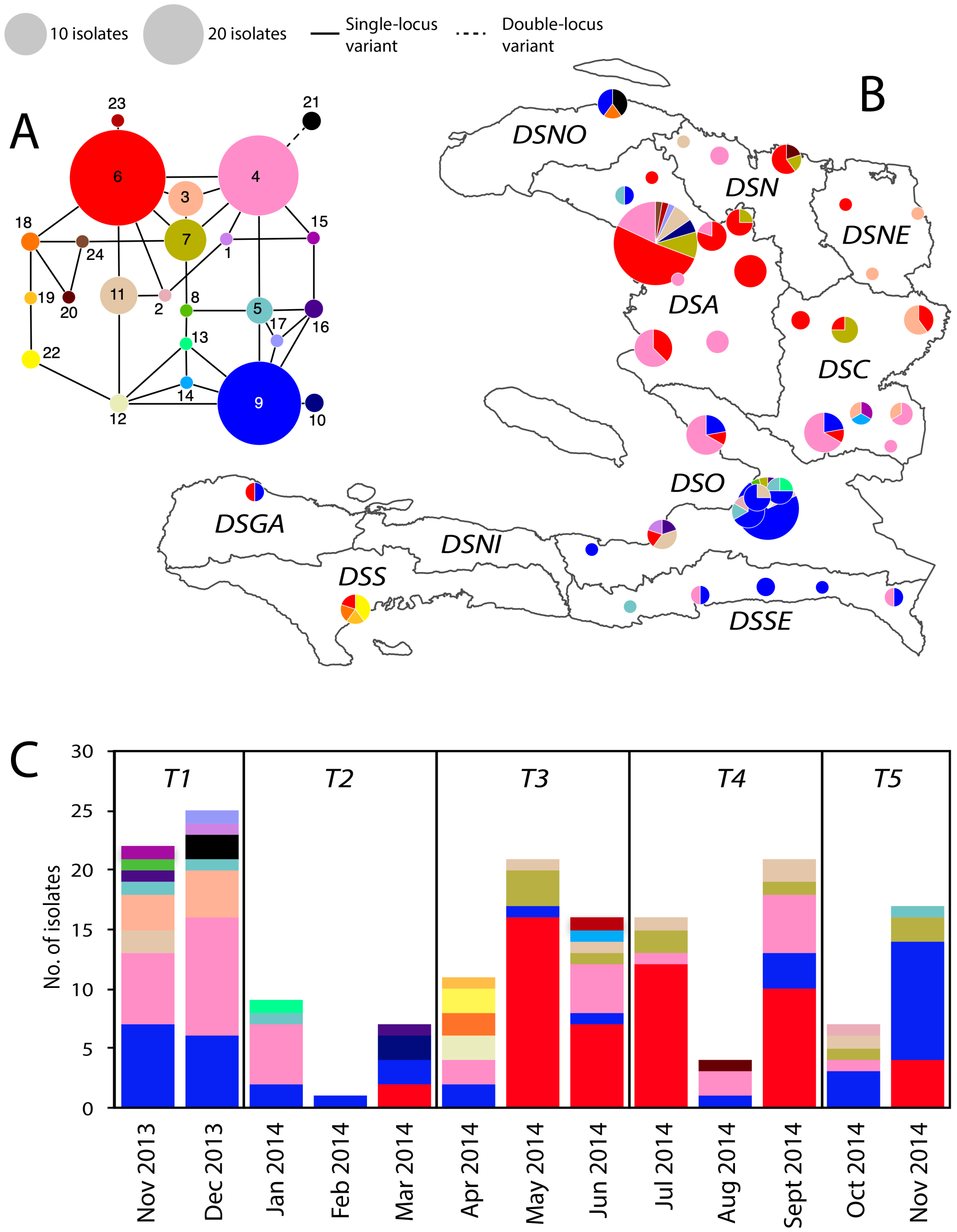

Overall, the differentiation index FST between the 5 trimesters exhibited a similar temporal structure to that between the 3 periods (S6 Table). Indeed, no significant genetic differentiation was observed between trimesters *T1* and *T2*, which nearly encompassed period *P1*, and between *T3* and T4, which nearly encompassed period *P2*. Conversely, populations *T1* and *T2* appeared genetically different

from *T3* and T4. Besides, *T3* and T4 were significantly different from *T5*, which nearly encompassed period *P3* (S6 Table).

Spatially, the differentiation index FST between the 10 departments exhibited a similar structure to that between the three zones (S6 Table). *DSO* population, which includes *PaP* zone, exhibited a strong genetic differentiation with *DSA*, *DSC*, *DSN* and *DSNE*, which nearly encompassed *North* zone. FST between *DSO* and departments covering the *South* zone (*DSSE*, *DSNI*, *DSS*, *DSGA*), were not significant, partly because of the small number of isolates (S6 Table).

Combining the five trimesters and the 10 departments, no significant genetic differentiation was observed between the five populations from the Ouest department (*DSO1*, *DSO2*, *DSO3*, *DSO4* and *DSO5*) (S6 Table). This matches with the low FST indexes between *PaP1*, *PaP2* and *PaP3*. Conversely, FST indexes remained high and often significant between populations from the Ouest department and populations from concomitant or preceding trimester in the northern departments. In particular, *DSO5* (population that includes the outbreak in Port-au-Prince) exhibited a strong genetic differentiation with *DSA3* and *DSA4* (populations aggregating isolates from Artibonite department during the two previous trimesters). Other genetic comparisons were limited by the small number of isolates in each population.

S6 Table – Genetic differentiation (fixation index FST) of V. cholerae O1 populations in Haiti between November 2013 and November 2014.

Trimester	No. isolates	FST *T2*	*T3*	*T4*	*T5*
T1	47	-0.001	0.224*	0.238*	0.053
T2	17		0.253*	0.302*	-0.014
T3	49			0.002	0.226*
T4	41				0.286*
T5	24				

Department	No. isolates	FST *DSA*	*DSC*	*DSN*	*DSNE*	*DSNO*	*DSSE*	*DSNI*	*DSS*	*DSGA*
DSO	50	0.472*	0.364*	0.424*	0.505*	0.187	-0.025	NA	-0.025	-0.146
DSA	69		0.07	-0.037	0.151	0.515*	0.543*	NA	0.543*	0.126
DSC	27			0.011	0.034	0.386	0.398	NA	0.398	0.009
DSN	8				0.049	0.346	0.501*	NA	0.501*	-0.004
DSNE	3					0.455	0.629	NA	0.629	0.152
DSNO	5						0.2	NA	0.2	-0.111
DSSE	8							NA	0	-0.045
DSNI	1								NA	NA
DSS	5									0.118
DSGA	2									

* significant FST

T1, Oct 2013 – Dec 2013; *T2*, Jan 2014 – Mar 2014; *T3*, Apr 2014 – Jun 2014; *T4*, Jul 2014 – Sept 2014; *T5*, Oct 2014 – Dec 2014

DSO, Ouest; *DSA*, Artibonite; *DSC*, Centre; *DSN*, Nord; *DSNE*, Nord-Est; *DSNO*, Nord-Ouest; *DSSE*, Sud-Est; *DSNI*, Nippes; *DSS*, South; *DSGA*, Grand'Anse

S6 Table (continued)
(Only populations with more than 2 isolates were included)

Trimester + Department	No. isolates	FST															
		DSO2	*DSO3*	*DSO4*	*DSO5*	*DSA1*	*DSA2*	*DSA3*	*DSA4*	*DSC1*	*DSC4*	*DSC5*	*DSN3*	*DSN4*	*DSNO1*	*DSSE1*	*DSS3*
DSO1	11	-0.128	-0.061	-0.022	-0.024	0.251	0.281	0.648*	0.559*	0.421*	0.539	0.292	0.395	0.47	0.12	-0.074	0.5
DSO2	6		-0.053	-0.016	-0.044	0.317	0.342	0.702*	0.622	0.485	0.614	0.3	0.449	0.493	0.133	-0.064	0.498
DSO3	10			-0.062	0.042	0.134	0.166	0.608*	0.493*	0.329	0.488	0.243	0.311	0.418	0.133	-0.106	0.514
DSO4	9				0.153	0.18	0.141	0.505*	0.393	0.294	0.37	0.117	0.212	0.291	0.129	0.006	0.441
DSO5	14					0.433	0.479	0.771*	0.709*	0.588*	0.743	0.545	0.641	0.7	0.235	-0.041	0.681
DSA1	9						0.003	0.615*	0.438*	0.117	0.519	0.253	0.163	0.449	0.436	0.194	0.682
DSA2	8							0.528*	0.323	0.069	0.395	0.174	0.04	0.325	0.404	0.254	0.646
DSA3	29								0.073	0.502*	-0.039	0.341	0.312	0.115	0.781*	0.73*	0.736*
DSA4	22									0.3*	-0.054	0.204	0.028	0.104	0.713	0.633*	0.719*
DSC1	15										0.388	0.206	0.015	0.351	0.561	0.428	0.711
DSC4	6											0.264	0.222	0.06	0.743	0.677	0.725
DSC5	5												-0.076	0.106	0.452	0.365	0.542
DSN3	4													0.095	0.567	0.471	0.667
DSN4	3														0.556	0.566	0.45
DSNO1	4															0.111	0.5
DSSE1	6																0.62
DSS3	4																

* significant FST
DSO1, Ouest department, *T1* trimester (Oct 2013 – Dec 2013); *DSA2*, Artibonite department, *T2* trimester (Jan 2014 – Mar 2014)…

S7 Appendix: Multiple component analysis and hierarchical classification of MLVA results

To confirm the MLG distribution analysis results and the FST differentiation statistics between populations, a classification of *V. cholerae* O1 isolates was performed according to the sizes of the six VNTRs. A hierarchical ascendant classification of the components of a multiple correspondence analysis (MCA) was performed using R [16] with the package FactoMineR, as previously described [17]. MCA was the first pre-processing step to explore the MLVA characteristics of the isolates, which takes into consideration relationships between variables. The coordinates of each variable in the first 20 principal components were used to perform a Hierarchical Ascendant Classification (HAC). This method provides classes according to the genetic profile using an objective non-supervised classification technique.

The HAC of MCA divided the isolates in three genetic clusters (S7 Figure, S7 Table). Distribution of these clusters among the *V. cholerae* O1 populations defined by the epidemic periods and the three zones is summarized in S4 Table. Cluster 1 grouped 116 of the 178 isolates. It mostly included isolates from the *North* zone throughout the three periods of the study and was the main cluster in these three populations. Cluster 2 grouped only nine isolates from the *North1*, *North2* and *South2* populations. Cluster 3 grouped 53 isolates, including 27 isolates from the *PaP* zone throughout the three periods, and was the main cluster in these three populations. Cluster 3 also included isolates from the *North1* and *South1* populations. This confirms the marked genetic differentiation between the *North* and *PaP* zones.

S7 Figure – (Panel A) Hierarchical Ascendant Classification (HAC), and (Panel B) Multiple correspondence analysis (MCA) of MLVA results of 178 *V. cholerae* O1 isolates in Haiti between November 2013 and November 2014. HAC was performed using the coordinates of each variable in the first 20 principal components of the MCA. In the factor map (Panel B), isolates were projected on the two first components of the MCA. Colors represent each of the three identified clusters. Dots in Panel B represent the 178 isolates.

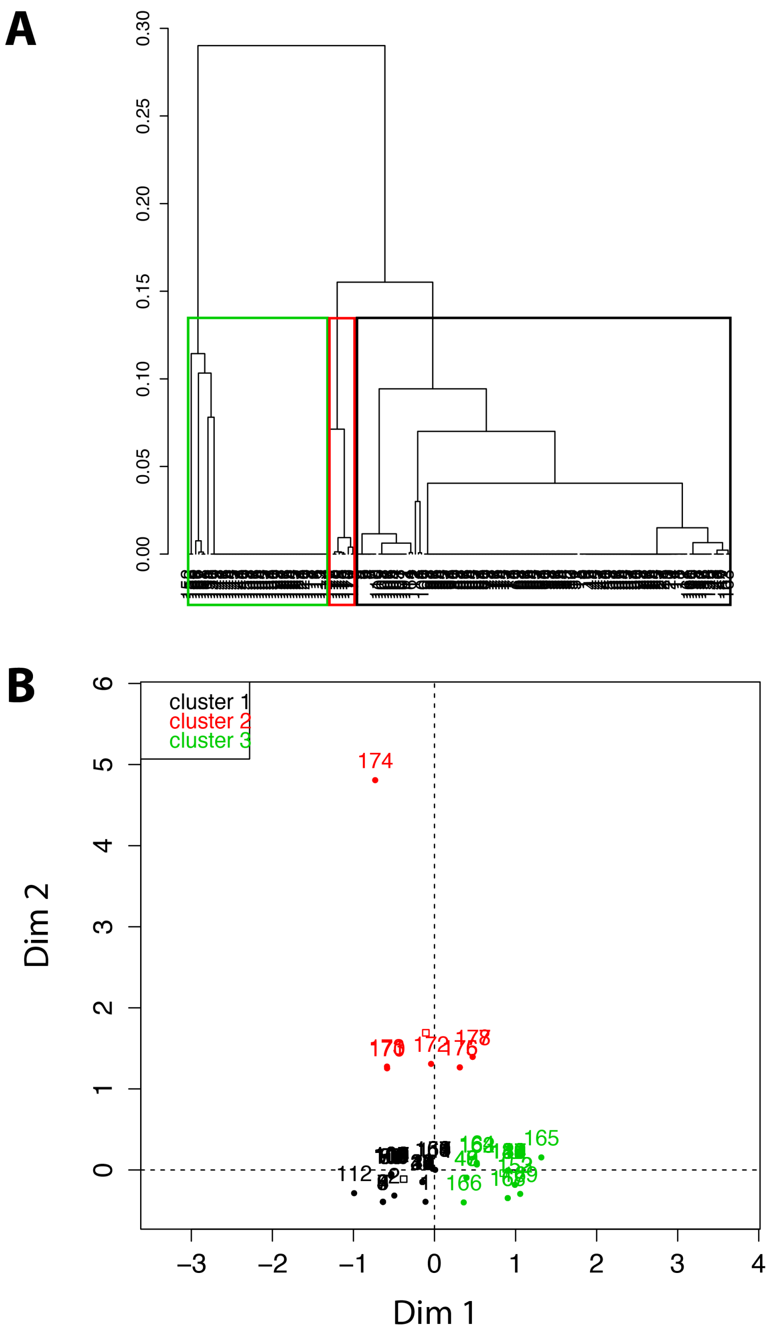

S7 Table – Distribution of the three clusters identified by HAC of MCA among the *V. cholerae* O1 populations defined by the three epidemic periods (*P1, P2* and *P3*) and the three zones (*North, PaP* and *South*)

	North1	*North2*	*North3*	*PaP1*	*PaP2*	*PaP3*	*South1*	*South2*	*South3*	Total
Cluster 1	26	58	19	1	0	4	5	1	2	116
Cluster 2	2	3	0	0	0	0	0	4	0	9
Cluster 3	10	3	4	5	10	12	8	0	1	53
Total	38	64	23	6	10	16	13	5	3	178

S8 Appendix: Multiple linear regression analysis of genetic, spatial and temporal distances between isolates

The structuration of the entire population was further assessed via multiple linear regression, which analyzed the relationship between the pairwise genetic distances of all 178 isolates and the associated spatial and temporal distances:

$$Genetic\_distance = Spatial\_distance + Temporal\_distance + Spatial\_distance{:}Temporal\_distance$$

The Genetic distance corresponded to the number of locus variants between each pair of isolates. The temporal distance corresponded to the lag between the sampling of isolates (number of days /365). The Euclidean distance was used to estimate the spatial distance from their coordinates. Analyses were computed using R version 3.2.1 for Mac [16], and the car package

The genetic distance between isolates appeared significantly correlated with their spatial distance (*p*-value <0.0001) and their temporal distance (*p*-value <0.0001) (S8 Table). This confirmed the marked genetic differentiation of *V. cholerae* O1 isolates in time and space between November 2013 and November 2014 in Haiti.

S8 Table – Relationship between the pairwise genetic distances of isolates and the associated spatial and temporal distances estimated by multiple linear regression analysis

All isolates (N=177) VS all previous isolates (N=177)	Regression coefficient (CI95%)	*p*-value
Spatial distance	2.85 (2.70-3.01)	<0.0001
Temporal distance	2.21 (2.00-2.45)	<0.0001

PaP3 isolates (N=16) VS All previous isolates (N=157)	Regression coefficient (CI95%)	*p*-value
Spatial distance	3.54 (2.98-4.19)	<0.0001
Temporal distance	0.89 (0.72-1.11)	0.3

Another regression model analyzed the distances between the 16 isolates sampled in the *PaP* zone during the *P3* period (*PaP3* population), and the 157 isolates previously sampled across the country. For this subset of isolates, the genetic distance was significantly associated with the spatial distance (*p*-value <0.0001), but not with the temporal distance (*p*-value = 0.3) (S8 Table). This result suggests that the *V. cholerae* O1 isolates from the outbreak in Port-au-Prince in late 2014 likely originated from a genetically stable autochthonous population rather than imported strains from distant areas of the country.

S9 Appendix: Bayesian clustering for spatial population genetics

In order to justify the choice of our populations (*North*, *Pap* and *South*) and assess its effects on our results, we completed our analysis with a Bayesian clustering algorithm for spatial population genetics implemented in the TESS program [18]. Using tessellations and Markov models, this program seeks population structure from individual multilocus genotypes sampled at distinct geographical locations without assuming predefined populations. Selection of the optimal number of clusters was based on the on the Deviance Information Criterion (DIC) which measures the prediction capabilities of the model.

The optimal number of clusters selected by the algorithm was 3, which fits with our initial choice to subdivide the population according to 3 zones. S9 Figure illustrates a remarkable match between the 3 identified clusters and our 3 populations of isolates (*North*, *PaP*, *South*).

S9 Figure – Bayesian clustering of multilocus genotypes grouped by *North*, *PaP* and *South* populations

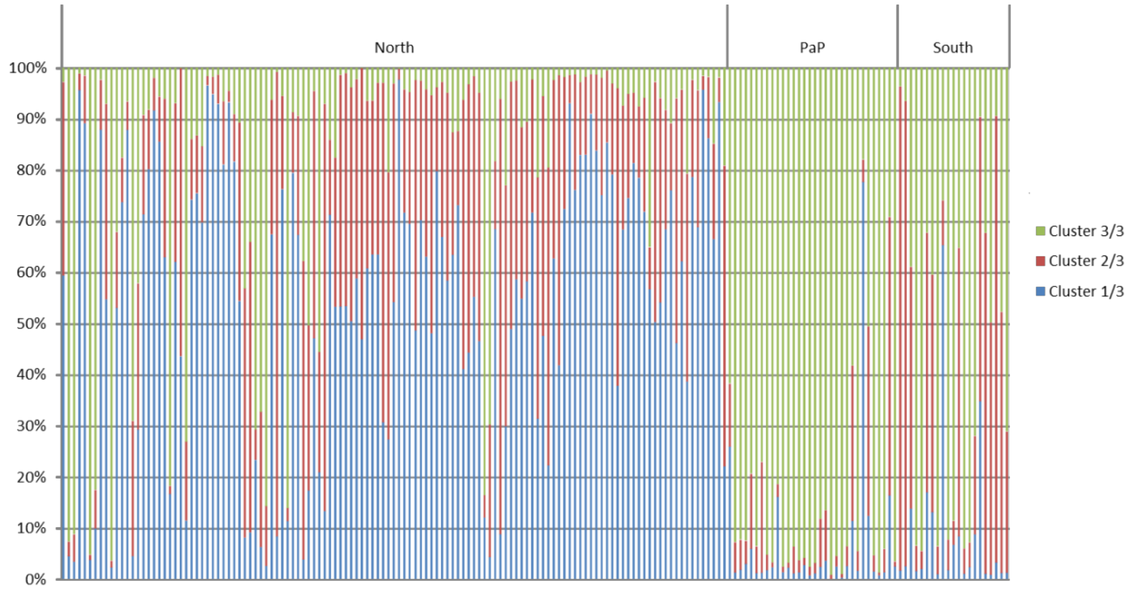

Appendix references

1. Kulldorff, M. & Information Management Services Inc. SaTScan - Software for the spatial, temporal, and space-time scan statistics. https://www.satscan.org/ (2018).

2. Azman, A. S., Rudolph, K. E., Cummings, D. A. T. & Lessler, J. The incubation period of cholera: A systematic review. *J Infect* **66**, 432–8 (2013).

3. Weir, B. S. & Cockerham, C. C. Estimating F-Statistics for the Analysis of Population Structure. *Evolution* **38**, 1358–1370 (1984).

4. Jost, L. G(ST) and its relatives do not measure differentiation. *Mol. Ecol.* **17**, 4015–4026 (2008).

5. Nei, M. Analysis of Gene Diversity in Subdivided Populations. *Proc Natl Acad Sci U S A* **70**, 3321–3323 (1973).

6. Hedrick, P. W. A standardized genetic differentiation measure. *Evolution* **59**, 1633–1638 (2005).

7. Ryman, N. & Leimar, O. G(ST) is still a useful measure of genetic differentiation - a comment on Jost's D. *Mol. Ecol.* **18**, 2084–2087; discussion 2088-2091 (2009).

8. Edelaar, P., Burraco, P. & Gomez-Mestre, I. Comparisons between QST and FST-how wrong have we been? *Molecular Ecology* **20**, 4830–4839 (2011).

9. Leng, L. & Zhang, D. E.-X. Measuring population differentiation using GST or D? A simulation study with microsatellite DNA markers under a finite island model and nonequilibrium conditions. *Mol. Ecol.* **20**, 2494–2509 (2011).

10. Leviyang, S. & Hamilton, M. B. Properties of Weir and Cockerham's Fst estimators and associated bootstrap confidence intervals. *Theor Popul Biol* **79**, 39–52 (2011).

11. Whitlock, M. C. Gst and D do not replace FST. *Molecular Ecology* **20**, 1083–1091 (2011).

12. Wang, J. Does GST underestimate genetic differentiation from marker data? *Molecular Ecology* **24**, 3546–3558 (2015).

13. Peter, B. M. Admixture, Population Structure, and F-Statistics. *Genetics* **202**, 1485–1501 (2016).

14. Jost, L. *et al.* Differentiation measures for conservation genetics. *Evol Appl* **11**, 1139–1148 (2018).

15. Goudet, J. FSTAT. https://www2.unil.ch/popgen/softwares/fstat.htm (2018).

16. R Core Team. R: A Language and Environment for Statistical Computing. R Foundation for Statistical Computing, Vienna, Austria. Available at: https://www.r-project.org/ (2018).

17. Lê, S., Josse, J. & Husson, F. FactoMineR: An R Package for Multivariate Analysis. *Journal of Statistical Software* **25**, (2008).

18. Chen, C., Durand, E., Forbes, F. & François, O. Bayesian clustering algorithms ascertaining spatial population structure: a new computer program and a comparison study. *Molecular Ecology Notes* **7**, 747–756 (2007).
